# Simultaneous determination of protoporphyrin IX and magnesium protoporphyrin IX in *Arabidopsis thaliana* and *Camellia sinensis* using UPLC-MS/MS

**DOI:** 10.1186/s13007-023-01008-y

**Published:** 2023-03-30

**Authors:** Chenyu Zhang, Chunlei Ma, Li Zhu, Mingzhe Yao

**Affiliations:** Key Laboratory of Biology, Genetics and Breeding of Special Economic Animals and Plants, Ministry of Agriculture and Rural Affairs, Tea Research Institute of the Chinese Academy of Agricultural Sciences, Hangzhou, 310008 China

**Keywords:** Mg-protoporphyrin IX, Protoporphyrin IX, UPLC-MS/MS, Chlorophyll biosynthesis, *Camellia sinensis*, *Arabidopsis thaliana*

## Abstract

**Backgrounds:**

Insertion of Mg^2+^ into protoporphyrin IX (PPIX) to produce magnesium-protoporphyrin IX (Mg-PPIX) was the first step toward chlorophyll biosynthesis, which not only imparts plants green pigmentation but underpins photosynthesis. Plants that blocked the conversion of PPIX to Mg-PPIX displayed yellowish or albino-*lethal* phenotypes. However, the lack of systematic study of the detection method and the metabolic difference between species have caused the research on chloroplast retrograde signaling controversial for a long time.

**Results:**

An advanced and sensitive UPLC-MS/MS strategy for determining PPIX and Mg-PPIX was established in two metabolic different plants, *Arabidopsis thaliana* (Columbia-0) and *Camellia sinensis* var. *sinensis*. Two metabolites could be extracted by 80% acetone (v/v) and 20% 0.1 M NH_4_OH (v/v) without hexane washing. Since the Mg-PPIX could be substantially de-metalized into PPIX in acidic conditions, analysis was carried out by UPLC-MS/MS with 0.1% ammonia (v/v) and 0.1% ammonium acetonitrile (v/v) as mobile phases using negative ion multiple reaction monitoring modes. Interestingly, it could be easier to monitor these two compounds in dehydrated samples rather than in fresh samples. Validation was performed in spiked samples and mean recoveries ranged from 70.5 to 916%, and the intra-day and inter-day variations were less than 7.5 and 10.9%, respectively. The limit of detection was 0.01 mg·kg^− 1^ and the limit of quantification was 0.05 mg·kg^− 1^. The contents of PPIX (1.67 ± 0.12 mg·kg^− 1^) and Mg-PPIX (3.37 ± 0.10 mg·kg^− 1^) in tea were significantly higher than in Arabidopsis (PPIX: 0.05 ± 0.02 mg·kg^− 1^; Mg-PPIX: 0.08 ± 0.01 mg·kg^− 1^) and they were only detected in the leaf.

**Conclusions:**

Our study establishes a universal and reliable method for determining PPIX and Mg-PPIX in two plants using UPLC-MS/MS. This procedure will facilitate studying chlorophyll metabolism and natural chlorophyll production.

## Background

**Scheme. 1:**
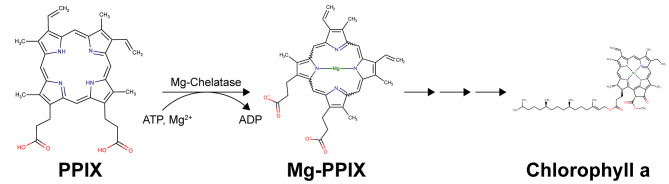
The schematic diagram for chlorophyll biosynthesis from protoporphyrin IX to chlorophyll a

Chlorophylls (Chls) underpin photosynthesis, which generates oxygen and fix carbon dioxide as carbohydrates that support life on Earth. The Chls biosynthetic pathway is a branch of tetrapyrrole biosynthesis [1], it begins with the insertion of Mg^2+^ into the protoporphyrin IX (PPIX) to form magnesium protoporphyrin IX (Mg-PPIX) that catalyze by magnesium chelatase complex (Scheme 1). Plants that blocked the conversion of PPIX to Mg-PPIX displayed chlorophyll-deficient or albino-*lethal* phenotypes [2]. In recent years, several studies reported that Mg-PPIX is not only an essential substrate for chlorophyll biosynthesis but also involved in the chloroplast retrograde signaling pathway [3–6]. In *Arabidopsis thaliana*, the mRNA level of nuclear-encoded plastid genes, such as *Lhcb1*, was depressed in wild-type plants when grown on norflurazon (NF) because of the chloroplast exert changes on nuclear gene expression [7]. Nevertheless, the *genome uncoupled* (*gun*) mutant, which showed disturbed retrograde signaling, retains a high level of *Lhcb1* when grown on NF [8–10]. Two *gun* mutants, *gun4* and *gun5*, were identified as involved in Mg-PPIX synthesis, which encoded a regulator of magnesium chelatase (MgCh) and ChlH, respectively [9, 11]. Moreover, a Chld knockout mutant and *chli1*/*chli1 chli2*/*chli2* double mutant also displayed the *gun* phenotype [3, 8], preliminarily suggesting that Mg-PPIX was a negative molecule in retrograde signaling (accumulation of Mg-PPIX triggered plastid to nucleus communication) [12]. However, several groups argued against this model, and the main controversy is the methodology for quantifying tetrapyrroles [13, 14].

Previous studies mainly employed spectrofluorometers or high-performance liquid chromatography (HPLC) coupled-spectrofluorometer to detect tetrapyrroles due to their photoreactive property [15]. The determination is made by employing specific wavelengths for excitation and emission, with concentrations being calculated by empirical formulae [16]. However, fluorescence detection can lead to the misidentification of related pigments having similar fluorescence properties. Besides, researchers established an in vivo system to visualize the Mg-PPIX in the cells using laser scanning spectroscopy, but this method could not absolutely quantify the Mg-PPIX content [17]. Michael and Alison tried to establish a method using HPLC-MS/MS to detect chlorophyll intermediates [18]. They found the signal of Mg-PPIX was poor in positive mode and formic acid could acidify Mg-PPIX if they wanted to increase ionization. More importantly, their method could not detect any chlorophyll intermediates in Arabidopsis no matter what with or without NF treatment, suggesting currently no available method can detect chlorophyll intermediates in plants sensitively and precisely. Based on their results, we speculated that the poor signal and the acidification of Mg-PPIX substantially influence the detection and a systematical methodology study should be conducted.

In this study, we developed a stable and sensitive UPLC-MS/MS method for the determination of both PPIX and Mg-PPIX in Arabidopsis and tea plants, which has abundant secondary metabolites that may impact the detection of tetrapyrroles [19]. The extraction procedures were comprehensively investigated including mobile phase (formic acid in water, formic acid in acetonitrile, formic acid, acetonitrile, ammonium in acetonitrile, ammonia, and ammonium acetate), extraction solvent (acetone, methanol and acetonitrile), sample treatment (fresh and dry sample), extraction way, extraction time, extraction temperature, clean-up time, and concentration influence. We found that the PPIX and Mg-PPIX contents in tea were 30 and 42 times higher than in Arabidopsis and they were only detected in the leaf. Overall, this study established a comprehensive and repeatable UPLC-MS/MS method for detecting PPIX and Mg-PPIX in different plants. This method could be used for validating the involvement of Mg-PPIX in retrograde signaling and discovering new regulation mechanism of the tetrapyrrole pathway.

## Results and discussions

### Optimization of the UPLC-MS/MS conditions

The previous study using LC-MS/MS to detect the PPIX and Mg-PPIX was conducted under ESI^+^ mode, while the response signal was weak [18]. Since the PPIX and Mg-PPIX have two carboxyl groups in the C and D pyrrole ring, the pool signal in positive mode was probably due to their acidic properties. We full scan dimethyl sulfoxide (DMSO)-standard solutions of the two compounds under ESI^+^ and ESI^–^ modes and found that the response value of PPIX and Mg-PPIX was markedly higher in ESI^−^ mode than ESI^+^. Thus, we choose ESI^−^ mode to conduct the following experiment. The multiple reaction monitoring (MRM)-associated parameters, such as con voltage and collision energy, were auto-optimized for the highest fragmented ions (Table 1).

**Table 1:** MS/MS parameters of PPIX and Mg-PPIX

Metabolite	Retention time (min)	Ion pair	Cone voltage (V)	Collision energy (eV)
PPIX	4.8	561.3/458.3	96	26
		561.3/473.3*		22
Mg-PPIX	4.9	583.3/480.2	98	28
		583.3/495.3*		28

Note: * indicates quantificational ion pairs

**Fig. 1:**
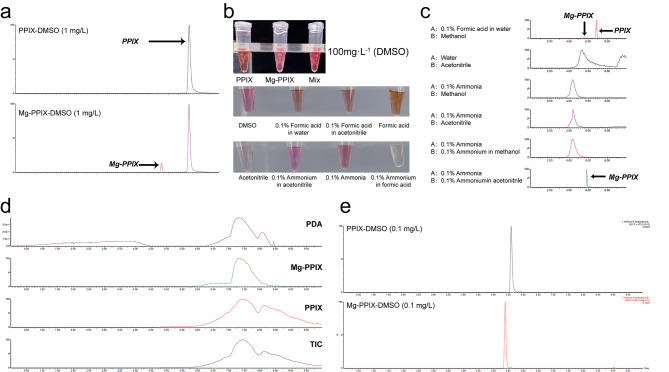
Mobile phase optimization of UPLC-MS/MS analysis for protoporphyrin IX (PPIX) and Mg-protoporphyrin IX (Mg-PPIX). a, UPLC-MS/MS analysis using 0.1% methanol in water (v/v) and formic acid as mobile phases. The 1% formic acid leads to Mg-PPIX demetalation to PPIX. b, Chromogenic reaction of Mg-PPIX with different solvents. DMSO, Dimethyl sulfoxide. c, UPLC-MS/MS analysis of Mg-PPIX using different mobile phases. d, UPLC-MS/MS analysis of PPIX and Mg-PPIX using 0.1% ammonia (v/v) and 0.1% ammonium in acetonitrile (v/v) as mobile phase

In the previous study, researchers added 2% formic acid to increase the ionization of Mg-PPIX but they found that it could induce demetalization of Mg-PPIX [18]. In this study, we also observed that few signals were accumulated in m/z 561.2 [M-H]^−^, when we directly injected Mg-PPIX standard (DMSO) into the tandem mass spectrometer using the mobile phases with formic acid. To avoid the demetalization of Mg-PPIX, firstly, we established a 10 min program for two compounds using methanol in water and formic acid as mobile phases. The result showed that approximately 80% of Mg-PPIX was converted to PPIX **(**Fig. 1a**)**. This result suggests that the Mg-PPIX could be converted to PPIX in formic acid condition while it had no significant influence on PPIX. To find optimum detection conditions, firstly we tested the chromogenic reaction of Mg-PPIX in eight organic solvents, including DMSO, formic acid in water (0.1%, v/v), formic acid in acetonitrile (0.1%, v/v), formic acid, acetonitrile, ammonium in acetonitrile (0.1%, v/v), ammonia (0.1%, v/v), and ammonium acetate (20 mM, pH 5.6). We found that the Mg-PPIX was rose red in DMSO, 0.1% ammonia water, and 0.1% ammonia in acetonitrile, while the color of Mg-PPIX was significantly changed in ammonium acetate, formic acid, formic acid water, formic acid acetonitrile, and acetonitrile, especially in ammonium acetate and formic acid **(**Fig. 1b**)**. Then, we used these solvents as mobile phases and monitored the status of Mg-PPIX in UPLC-MS/MS. The results showed that the Mg-PPIX peak was well-retained (without demetalization) using ammonia and ammonium acetonitrile as mobile phases **(**Fig. 1c**)**, whereas the signals of Mg-PPIX were extremely weak in other mobile phases. We particularly investigated the status of PPIX and Mg-PPIX using ammonium acetate and methanol as mobile phases, because it has been reported in numerous studies [3, 18]. However, we found strong cross-talk among different signal channels, and even no peak was observed under the current condition **(**Fig. 1d**)**. Overall, our study indicated that acidic mobile phase(s) can easily induce the demetalization of Mg-PPIX, hence ammonia and ammonium acetonitrile served as mobile phases were rational and optimal **(**Fig. 1e**)**.

### Optimization of the pre-treatments

**Fig. 2:**
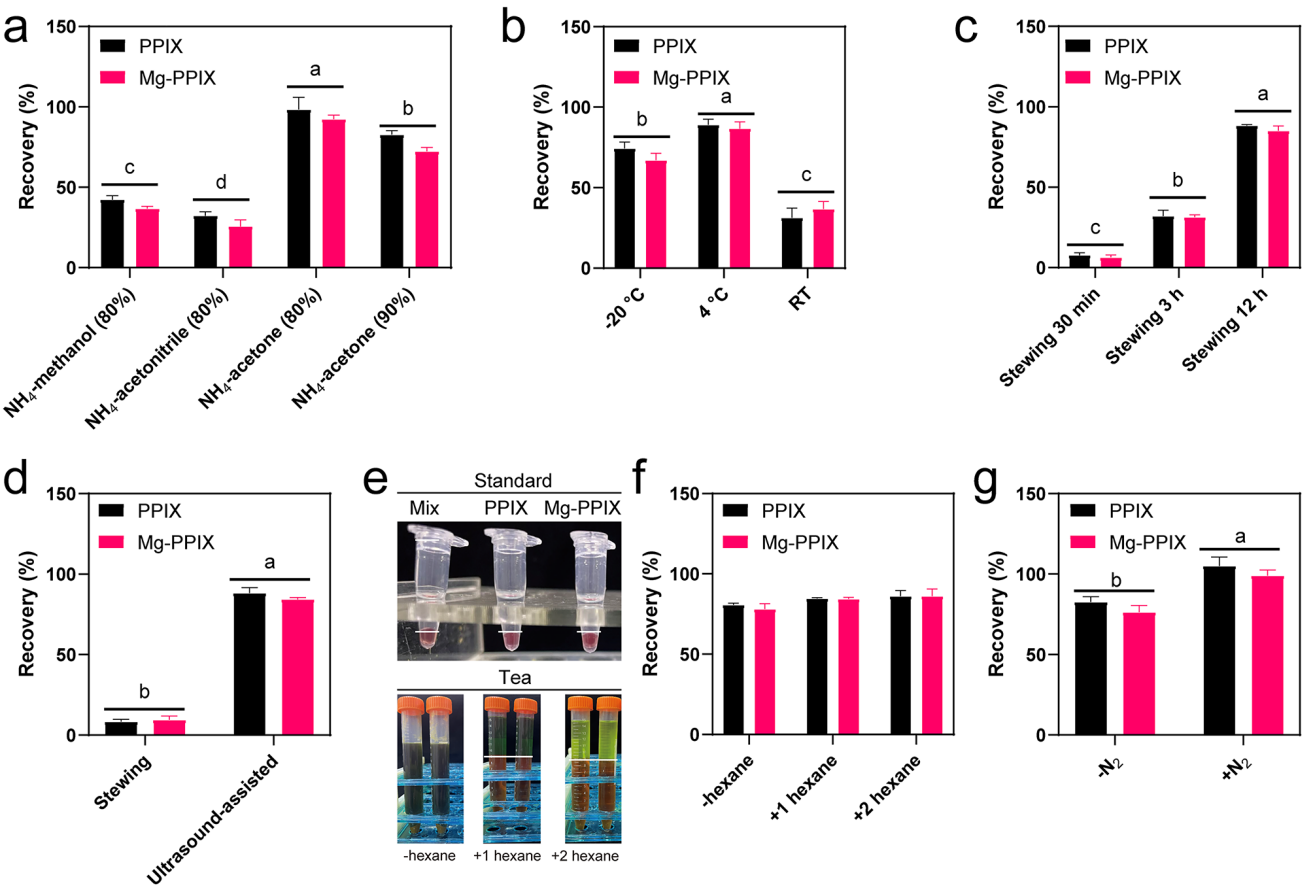
Improvement for pre-treatment steps of PPIX and Mg-PPIX under different conditions. a, extraction solvents; b, extraction temperatures; c, extraction time; d, extraction way; e and f, impurity removal times; g, concentration way

Extraction is one of the most critical steps in the pre-treatment for high recoveries. Since the tetrapyrroles have weak polarity (only dissolve in organic solvent) and high solubility in acetone, most studies used methanol and ammonia acetone as the extraction solvents for PPIX and Mg-PPIX [8, 13, 14]. However, the extraction procedures, such as the ratio between ammonia and acetone, extraction time, temperature, and way have not been reported. To test their effect, we spike matrix-standard solutions of PPIX and Mg-PPIX (1 mg·kg^− 1^) into the blank sample (liquid nitrogen grinding) and evaluate the recoveries under different conditions. The recoveries that used acetone as an extract were significantly higher than methanol and acetonitrile **(**Fig. 2a**)**. Besides, the recovery of 80% ammonium acetone (v/v) was higher than 90% ammonium acetone (v/v). Three temperatures, -20 °C, 4 °C, and room temperature (RT), were tested and the highest and the lowest recoveries were obtained when the extraction temperatures were in 4 °C and RT, respectively **(**Fig. 2b**)**. We observed that the recoveries of both PPIX and Mg-PPIX was significantly increased after stewing 3 and 12 h compared to the 30 min **(**Fig. 2c**)**. Moreover, we found that the recoveries exhibited significantly difference between ultrasound-assisted extraction and stewing extraction, and the recoveries was approximately over 90% using ultrasound-assisted extraction for 30 min **(**Fig. 2d**)**. In the previous studies, several experiments added hexane to purification the acetone extract [20], because it can clean-up chlorophyll from acetone (up: hexane, low: acetone) and the spectrophotometer was highly sensitive to the pigments **(**Fig. 2e**)**. We found that the recoveries were increased after clean-up without significant change **(**Fig. 2f**)**. Moreover, previous studies used nitrogen-blow to concentrate extract [20], but this procedure may increase the interference from non-target analytes, especially for perennial plants, such as tea plants. The result showed that nitrogen blow can significantly increase the response value **(**Fig. 2g**)**.

**Fig. 3:**
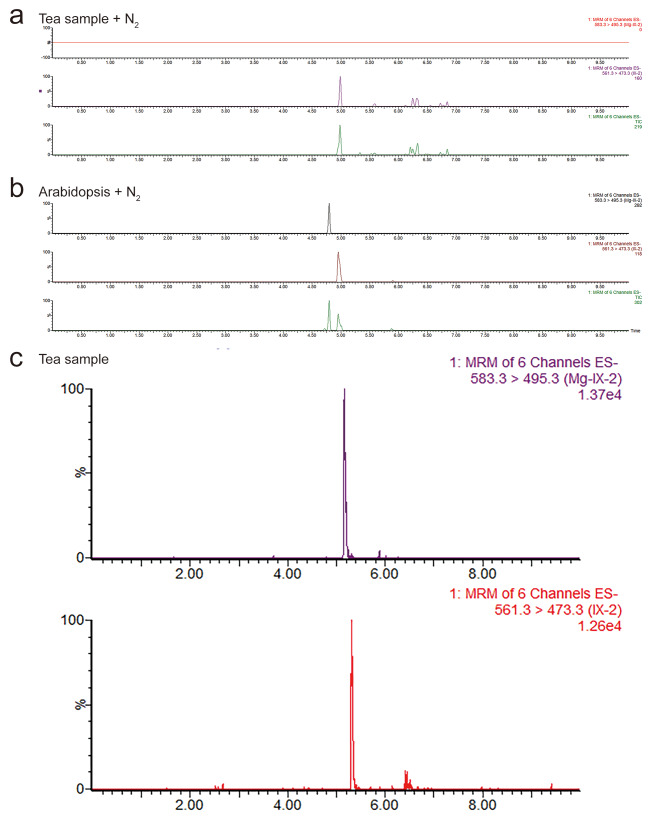
The MRM chromatogram of PPIX and Mg-PPIX in Arabidopsis and tea plant. a, in tea mature leaf; b, in Arabidopsis mature leaf; c, in tea tender leaf

Since chlorophyll biosynthesis is a primary metabolic pathway for plants, the PPIX and Mg-PPIX should be easily detected after we optimize the extraction procedure. To our confusion, we could only detect PPIX, rather than Mg-PPIX, in the fresh tea sample, no matter how many times it is concentrated **(**Fig. 3a**)**. Also, we monitored a trace amount of PPIX and Mg-PPIX in fresh Arabidopsis rosette leaf (two week old) after concentrating **(**Fig. 3b**)**. To eliminate operating errors, we spiked standards before and after grinding but the result showed no significant difference. Finally, we have to speculate that the Mg-PPIX was demetalized at the moment of grinding due to the acidic tea matrix (pH = 6.25 in water). Therefore, we attempted the fixing methods of liquid nitrogen grinding, microwave drying, and freeze drying. Results showed that the Mg-PPIX can be detected in both microwave drying and freeze-drying tea samples but not in liquid nitrogen grinding tea samples, and the responsive value of PPIX and Mg-PPIX in freeze drying sample (tender leaf) was very high so it could save nitrogen-blow procedure **(**Fig. 3c**)**.

### Method validation

**Fig. 4:**
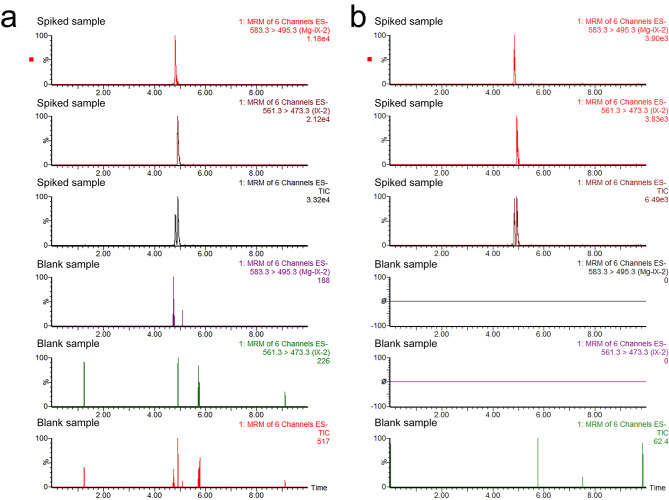
The MRM chromatogram of the blank and spiked sample. a, tea leaf; b, Arabidopsis

The MRM chromatograms of the blank and spiked samples of both tea and Arabidopsis are shown in Fig. 4, they were separated in the baseline. The solvent and matrix-matched linear equations are summarized in (Table 2) with a correlation coefficient > 0.99. The matrix effect (ME) was also calculated according to the previous study [21] and the ME of PPIX were 19.49% and 12.00% in tea and Arabidopsis, respectively, the ME of Mg-PPIX were 9.40% and 2.32% in tea and Arabidopsis. These results suggest that less ME was found in Arabidopsis than in tea, and the ME did not significantly interfere with the detection. The limitation of detection (LOD) and limitation of quantification (LOQ) was determined based on the S/N ratio > 3 and S/N > 10, respectively (Table 2). The LOD of both tea and Arabidopsis was 0.001 mg·kg^− 1^, and the LOQ of tea and Arabidopsis range from 0.003 to 0.005 mg·kg^− 1^.

**Table 2:** Linear Calibration Range, Standard curve, LOD, and LOQ of PPIX and Mg-PPIX in Arabidopsis and tea plants

Matrix	Metabolite	Linear range(mg/kg)	regression equitation (Matrix)	Correlation coefficient, *R*^*2*^	Matrix effect (%)	LOD (mg/kg)	LOQ (mg/kg)
Arabidopsis	PPIX	0.005-1.00	Y = 621.771X + 5.68201	0.999984	12.00%	0.001	0.005
	Mg-PPIX	0.005-1.00	Y = 530.88X-6.70815	0.994277	2.32%	0.001	0.004
Tea plant	PPIX	0.005-5.00	Y = 568.914X-1.46632	0.999062	19.49%	0.001	0.005
	Mg-PPIX	0.005-5.00	Y = 567.683X + 9.00217	0.994476	9.40%	0.001	0.003

The accuracy and precision of this procedure were evaluated by determining the recovery as well as intra-day and inter-day relative standard deviations (RSD). Each process was conducted at two different levels (0.1 and 0.01 mg·kg^− 1^) with six replicates on three separate days. Mean recoveries ranged from 70.5 to 86.4% for PPIX and from 87.6 to 91.6% for Mg-PPIX with the intra-day RSD < 7.5% and inter-day RSD < 10.9%, respectively (Table 3).

**Table 3:** Accuracy and Precision of PPIX and Mg-PPIX in Arabidopsis and tea plant

Matrix	Metabolite	Spiked concentration (mg/kg)	Mean recovery (%)	Intra-day RSD% (*n* = 6)	Inter-day RSD% (*n* = 18)
Arabidopsis	PPIX	0.1	85.5	5.7	8.7
		0.01	86.4	7.5	5.9
	Mg-PPIX	0.1	89.5	4.6	10.9
		0.01	91.6	6.6	9.8
Tea plant	PPIX	0.1	74.8	7.1	6.2
		0.01	70.5	7.5	6.2
	Mg-PPIX	0.1	88.1	5.9	3.5
		0.01	87.6	3.2	6.2

### Determination of PPIX and Mg-PPIX in tea and arabidopsis

The developed analytical method was applied for the determination of PPIX and Mg-PPIX in Arabidopsis thaliana (Col-0) and tea (‘Longjin43’). Four positive samples were detected in tender leaf samples with concentrations ranging from 0.05 to 0.08 mg·kg^-1^ (DW) in Arabidopsis and 1.67–3.37 mg·kg^-1^ (DW) in tea (Table 4). The level of Arabidopsis PPIX and Mg-PPIX in our study was similar with the previous study (PPIX ≈ 0.022 mg·kg^-1^, FW and Mg-PPIX ≈ 0.017 mg·kg^-1^, FW ) [13]. The level of PPIX in tea was approximately 30 times higher than in Arabidopsis and the level of Mg-PPIX in tea was approximately 42 times higher than in Arabidopsis. This result indicates that the content of tetrapyrroles varied significantly between species. As to mature leaves, we only detect a trace amount of Mg-PPIX in Arabidopsis (Table 4). To further verify this development procedure, we detect the PPIX and Mg-PPIX in different tissue of tea plants (‘Zhongcha 108’), including tender leaf, mature leaf, stem, root, seed, and episperm. The results were mostly in agreement with tea (‘Longjin43’), indicating PPIX and Mg-PPIX at tender leaf were 1.31 ± 0.15 mg·kg^-1^ and 2.99 ± 0.09 mg·kg^-1^, respectively (Table 4). However, we also detect the PPIX and Mg-PPIX in the mature leaf that was only 0.12 ± 0.01 mg·kg^-1^ and 0.14 ± 0.03 mg·kg^-1^, respectively. As to other tissues, the compounds were not detected in all samples, which is probably because of the shortage of equipment sensitivity. Moreover, whether this method can detect monocotyledonous plants and photosynthetic microorganisms remains to be studied.

**Table 4:** Concentrations of PPIX and Mg-PPIX in different species and tissues

Sample	Type	PPIX (mg/kg, DW)	Mg-PPIX (mg/kg, DW)
Arabidopsis	Tender leaf	0.05 ± 0.02	0.08 ± 0.01
	Mature leaf	n.d.	0.03
Tea plant (LJ43)	Tender leaf	1.67 ± 0.12	3.37 ± 0.10
	Mature leaf	n.d.	n.d.
Tea plant (ZC108)	Tender leaf	1.31 ± 0.15	2.99 ± 0.09
	Mature leaf	0.12 ± 0.01	0.14 ± 0.03
	Stem	n.d.	n.d.
	Root	n.d.	n.d.
	Seed	n.d.	n.d.
	Episperm	n.d.	n.d.

Note: n.d. indicates non-detection; DW, dry weight

## Conclusion

A quantitative and confirmatory UPLC-MS/MS procedure for the determination of PPIX and Mg-PPIX in Arabidopsis and tea was developed in this study. Taking the pre-treatment step together, the extraction procedures were fully investigated. The concentration of target compounds was significantly different in the two species, which probably because of the tea plant was a metabolism-abundant species [22]. This proposed analytical procedure will benefit not only the functional validation of magnesium chelatase subunits but the research of chloroplast-nucleus retrograde signaling, which is essential for discovering the chloroplast biogenesis and coordination of the expression of nuclear and chloroplast genes [5]. In future, we aim to improve this method by detecting more species, including monocotyledons and photosynthetic microorganism, and establish a high-throughput targeted metabolome method that can simultaneously detect all known metabolites in chlorophyll biosynthesis pathways.

## Materials and methods

The UPLC-MS/MS was carried out with ACQUITY UPLC H-Class/Xevo TQ-S microsystem (Waters, USA). HPLC-grade acetonitrile, methanol, and acetone were purchased from Fisher Scientific, Inc. (Ontario, Canada). Ammonium acetate (HPLC-grade) was purchased from Solarbio (Beijing, China). Standards of protoporphyrin IX (PPIX; CAS: 53-12-8) and Mg(II) protoporphyrin IX dipotassium salt (Mg-PPIX; CAS: 35979-27-2) were purchased from Frontier Scientific, Inc., (Utah, USA). *Arabidopsis thaliana* (Col-0) and *Camellia sinensis* (L.) O. Kuntze ‘Longjin 43’ and ‘Zhongcha 108’ were used in this study. The rosette leaves of Arabidopsis (two weeks) and the second and third leaves of the tea plant were collected in September 2022.

### Plant growth conditions

The seeds of *Arabidopsis thaliana* (Col-0) were directly germinated in peat pellet (Jiffy Products International BV, Norway) and grew in the incubator (Ningbo Ledian instrument manufacturer Co., LTD, Ningbo, China) under a long-day photoperiod (16 h light/8 h dark) with a light intensity of 150 µmol m^− 2^ s^− 1^ and relative humidity of 60% at 21 °C ± 2 °C. The tea plants (cv. ‘Longjin43’ and cv. ‘Zhongcha 108’) were cultivated in the National Germplasm Hangzhou Tea Repository of TRICAAS, China.

### Preparation of standard solution

The standards of PPIX and Mg-PPIX (10 mg) were dissolved in DMSO (10 mL) to make up the stock solution (1000 mg·L^− 1^) and stored at -20 °C. The mixed stock solution (10 mg·L^− 1^) was prepared by adding 0.1 mL stock (1000 mg·L^− 1^) per standard and volume in 10 mL DMSO. The working solution was prepared using DMSO and diluted to 5, 1, 0.5, 0.1, 0.05, 0.01, and 0.005 mg·L^− 1^.

### PPIX and Mg-PPIX extraction from plants

The dry sample (100 mg) was ground using TissueLyser II (Qiagen, German) and ultrasound-assisted extraction (42 K HZ) with 10 mL acetone:0.1 M NH_4_OH (8:2, v/v) for 30 min at 4 °C using ultrasonic cleaning machine (Branson, USA). The extract was centrifuged at 4,000 rpm at 4 °C for 10 min and the supernatant was transferred into a new centrifuge tube. Adding equal-volume hexane and centrifuging (4,000 rpm) for 5 min at 4 °C. Removed the supernatant carefully and the tetrapyrroles were retained in the acetone phase. The remaining extract was filtered by a Millipore filter (0.22 μm) before detection for sterilization.

### Ultra-performance liquid chromatography/tandem mass spectrometry (UPLC-MS/MS) analysis

Chromatographic separation was performed with an ACQUITY UPLC BEH C18 Column (3.0 mm × 150 mm ×1.7). The solvent elution program was as follows: 0–1 min 98% solvent A and 2% solvent B; 1–8 min 0% solvent A and 100 solvent B; 8.0–10 min 98% solvent A and 2% solvent B. Column temperature: 40 °C; Injection volume: 5 µL.

The mobile phase A and B was 0.1% aqueous ammonia (v/v) and 0.1% ammonium acetonitrile (v/v). The flow rate was 0.4 mL·min^− 1^, and the absorption spectra of PPIX and Mg-PPIX were detected by a photodiode array detector (PDA) in the range of 380–420 nm.

The mass spectra were recorded according to the optimal parameter, ESI^−^ source temperature, 400 °C; capillary voltage, 3.6 kV; desolvation gas flow, 800 L/h; cone gas flow, 50 L/h; collision gas: Ar; carrier gas, N_2_.

### Recovery and matrix effect assay of the spiked sample

Adding mixed standards into the sample till 1 mg·kg^− 1^. The extraction and detection processes were identical to the abovementioned method. The recovery and relative standard deviation were calculated by: the peak intensity of matrix-standard to solvent-standard.

The standards (0.1 mg·kg^− 1^ and 0.01 mg·kg^− 1^) were added to the sample before extraction, and the matrix effect was calculated as follows: $\text{M}\text{E}=\frac{\text{k}\text{m}\text{a}\text{t}\text{r}\text{i}\text{x}-\text{k}\text{s}\text{o}\text{l}\text{u}\text{t}\text{i}\text{o}\text{n}}{\text{k}\text{s}\text{o}\text{l}\text{u}\text{t}\text{i}\text{o}\text{n}}\times 100\%$

### Statistical analysis

The one-way ANOVA and Student′s t-test were performed using GraphPad Prism 8.0.1. (San Diego, CA, USA) to evaluate the difference, and *p* < 0.05 was considered statistically significant. All data were presented as the mean values ± standard deviation based on three independent biological replicates.

## Data Availability

Not applicable.
